# Supplementary material for: Bmal1 Regulates Prostate Growth via Cell-Cycle Modulation
Source: Int J Mol Sci. 2022 Sep 24;23(19):11272. doi: 10.3390/ijms231911272 (PMC9569634; doi:10.3390/ijms231911272)
Supplement: Supplementary file 1 [file ijms-23-11272-s001.zip › ijms-1911167-supplementary.pdf]

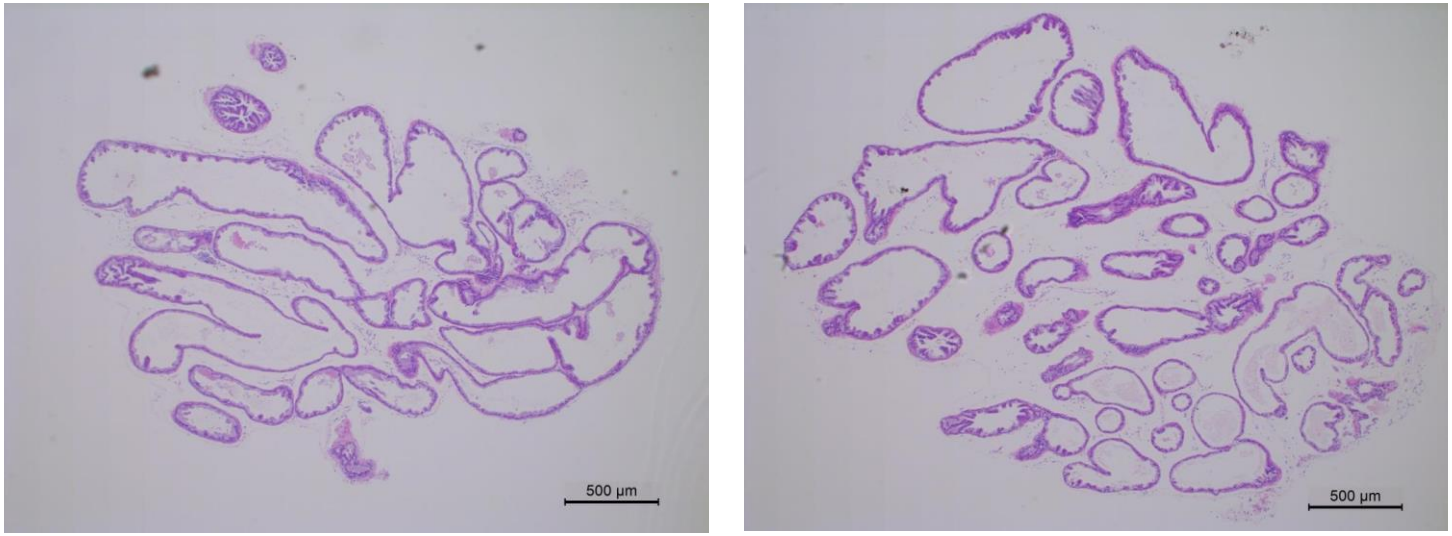

Bmal1$^{fx/fx}$ *Bmal1*$^{fx/fx}$ *– Cre*

Figure S1: Histological analysis of ventral prostates in *Bmal1*$^{fx/fx}$ mice and *PbsnCre+; Bmal1*$^{fx/fx}$ mice. Sections were stained with hematoxylin and eosin (H&E).

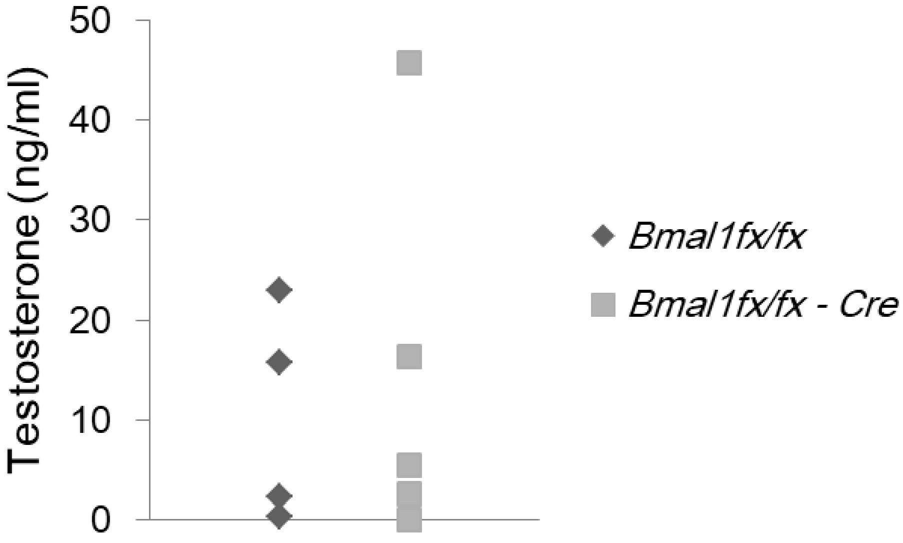

Figure S2: Serum testosterone concentration of *PbsnCre+; Bmal1*$^{fx/fx}$ mice (*N*=5) and *Bmal1*$^{fx/fx}$ mice (*N*=4).

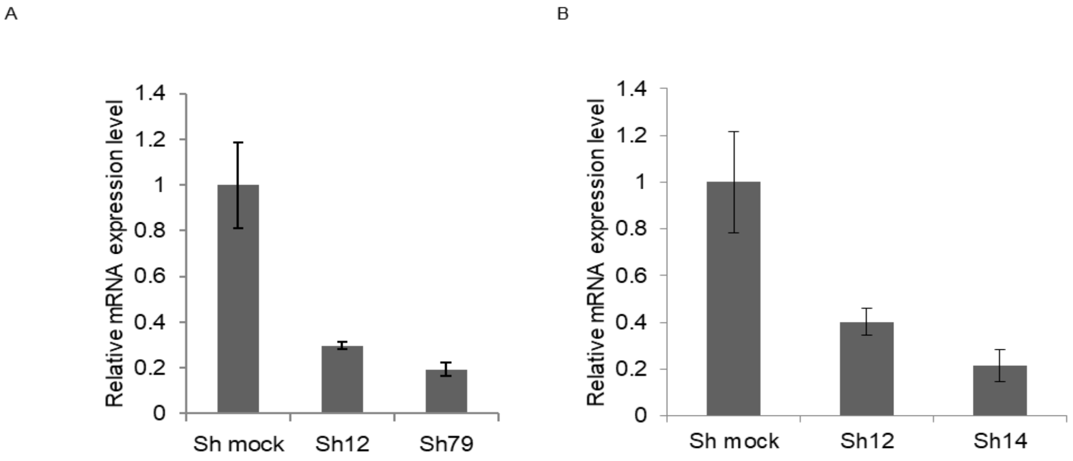

Figure S3: The levels of *BMAL1* expression in *BMAL1*-knockdown cells. (A) prostate epithelial cells, RWPE-1. (B) prostate stromal cells, WPMY-1.

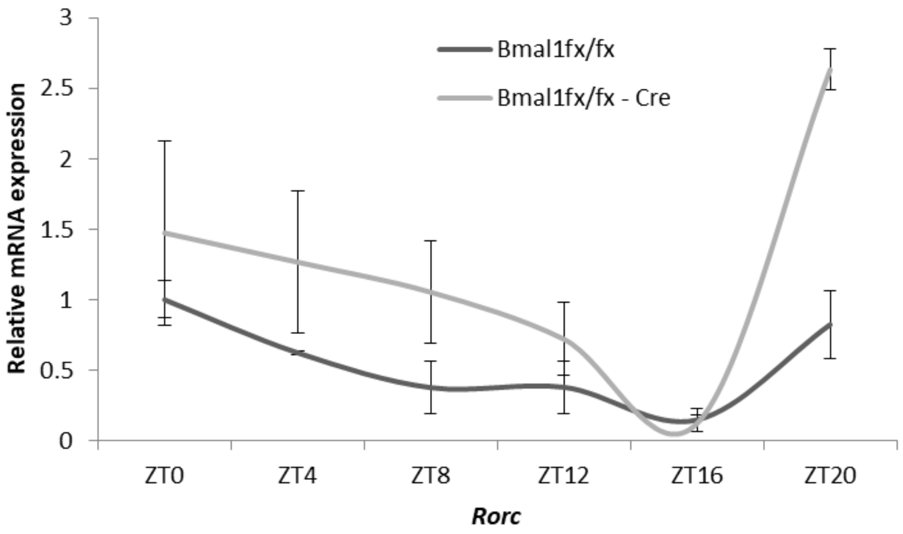

Figure S4: Oscillation of *Rorc* mRNA in *Bmal1*$^{fx/fx}$ mice and increased expression of *Rorc* mRNA in mouse prostates from pBmal1 KO mice. Oscillation of *Rorc* in mouse dorsolateral prostates under LD condition (*N*=3 for each time point). P-values with Cosinor analysis in *Rorc* were 0.091 in *Bmal1*$^{fx/fx}$ mice and 0.366 in pBmal1 KO mice, respectively.

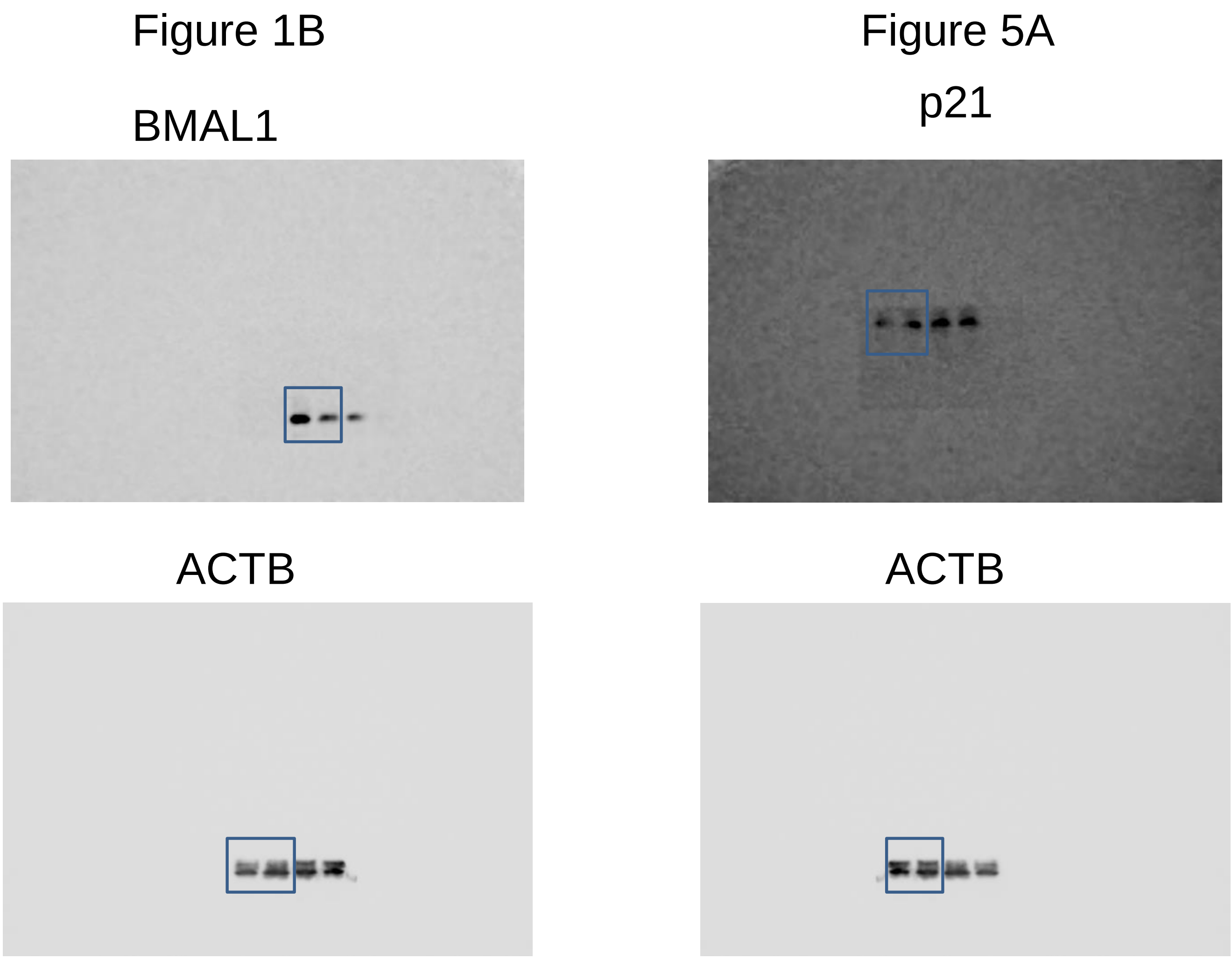

Figure S5: Full-length unedited blots for Figure 1B and 5A

Table S1. Primers for real-time quantitative PCR.

Species	Gene name		Primer sequences	Amplicon size (b.p.)
Human	*Bmal1*	Forward	GAAGACAACGAACCAGACAATGAG	86
		Reverse	GGTTGTGGAACTACATGAGAATGC	
	GAPDH	Forward	GAAGGTGAAGGTCGGAGTC	226
		Reverse	GAAGATGGTGATGGGATTTC	
Mouse	*Bmal1*	Forward	CCAAGAAAGTATGGACACAGACAAA	81
		Reverse	GCATTCTTGATCCTTCCTTGGT	
	Cdkn1a	Forward	GCAGACCAGCCTGACAGATTT	211
		Reverse	GAGAGGGCAGGCAGCGTAT	
	Rorc	Forward	CGACTGGAGGACCTTCTACG	94
		Reverse	GCTCCCACATCTCCCACA	
	Rn18S	Forward	ACTCAACACGGGAAACCTCA	123
		Reverse	AACCAGACAAATCGCTCCAC	
